# Comparison of screening tests in the evaluation of cognitive status of patients with epilepsy

**DOI:** 10.1590/1980-57642021dn15-010016

**Published:** 2021

**Authors:** Mayla Cristine de Souza, Carolina Oliveira de Paulo, Larissa Miyashiro, Carlos Alexandre Twardowschy

**Affiliations:** 1 Medicine School, Pontifícia Universidade Católica do Paraná – Curitiba, PR, Brazil.

**Keywords:** epilepsy, cognition, memory disorders, epilepsia, cognição, transtornos da memória

## Abstract

Epilepsy, a chronic neurological condition which is associated with neurobiological and psychosocial changes, affects 0.5 to 1% of the world's population, presenting in most cases a deficit in reasoning, memory and attention.

**Objective:**

To contribute to the implementation of screening strategies for cognitive decline and memory deficits in patients with epilepsy.

**Methods:**

Two questionnaires, MMSE and MoCA, were used in this cross-sectional and observational study. Fifty-four patients diagnosed with different types of epilepsy (55% refractory) were assessed; they were all over 18 years old, of both genders, with autonomy to answer the questionnaire. They were followed exclusively at an outpatient clinic of the Neurology Service Department, specialized in epilepsy, which is part of the tertiary healthcare level of the Brazilian Unified Health System (SUS).

**Results:**

The final sample consisted of 54 patients. There was a significant correlation (p<0.001) between the scores of both tests, indicating that low values in the MMSE score also corresponded to low values in the MoCA score. Sensitivity was 90% (ROC curve adjusted) and 87.5% of the patients with a normal score in the MMSE test obtained alterations in the MoCA scores. None of them showed a low MMSE score with a normal MOCA score. The Spearman correlation coefficient was 0.80. Also, there was a significant relationship between both immediate memory and delayed recall memory and the type of seizure (p<0.03) and level of schooling (p<0.001), respectively.

**Conclusion:**

The MoCA is a well-suited test to be performed in epilepsy patients to evaluate their cognition as it seems more extensive and complete compared to MMSE.

## RESUMO.

A epilepsia, condição neurológica crônica associada a alterações neurobiológicas e psicossociais, afeta de 0,5 a 1% da população mundial. Na maior parte dos casos, há redução de raciocínio, memória e atenção.

**Objetivos:**

Contribuir para a implementação de estratégias de rastreio de declínio cognitivo e distúrbios na memória nos pacientes com epilepsia.

**Métodos:**

Estudo transversal observacional de 54 pacientes diagnosticados com epilepsia de diversos tipos (55% refratários) e com idade superior a 18 anos, de ambos os sexos, com autonomia para responder o questionário e em acompanhamento exclusivamente pelo Sistema Único de Saúde (SUS) em um ambulatório especializado em epilepsia, do serviço de neurologia, que faz parte do nível terciário de atenção à saúde. Foram aplicados dois questionários: o MEEM e o MoCA.

**Resultados:**

Amostra final de 54 pacientes. Encontrou-se uma correlação significativa (p<0,001) entre os escores dos dois testes, o que significa que valores baixos do escore MEEM correspondem a valores baixos do escore MoCA. Sensibilidade de 90% (curva ROC ajustada). Verificou-se que dentre os pacientes considerados normais no MEEM, 87,5% deles obtiveram escore com alterações por meio do teste de rastreio MoCA. Não se obteve nenhum caso de escore no MEEM baixo com pontuação no MoCA normal. O coeficiente de correlação Spearman foi 0,80. Há relação significativa da memória imediata e evocação tardia com o tipo de crise (p<0,03) e escolaridade (p<0,001), respectivamente.

**Conclusão:**

Torna-se pertinente a adição do teste MoCA para rastreio cognitivo em pacientes com epilepsia por ser um instrumento mais extenso e preciso, minimizando as chances de “falsos-negativos” quando comparado ao MEEM.

## INTRODUCTION

Epilepsy is a chronic, electrical brain disorder with neurobiological, social, cognitive and psychological consequences.^1^ ^,^ ^2^ Epilepsy patients not only tend to have lower social interaction and less employment opportunities, but they also tend to have emotional distress in comparison to other chronic diseases.^3^ ^,^ ^4^ In addition, the majority of epileptic patients are more prone to have a lower cognitive performance than a control group without the disease.^4^ ^,^ ^5^

Both focal and generalized epilepsy can cause deficits in memory, language and motor functions. Patients with temporal lobe epilepsy or focal onset showed more memory deficits in comparison to those with extratemporal epilepsy.^6^ Also, deficits in attention and memory were detected in 30% of newly diagnosed patients with cryptogenic epilepsy.^5^ Additionally, in a study with a series of neuropsychological tests with the aim of assessing both memory and psychomotor skills, about 53% of newly diagnosed and untreated epileptic patients had at least abnormal test scores in comparison to the mean of the control group.^7^

Neuropsychological evaluation is mandatory in the follow-up of patients with this condition, due to their cognition declines,^8^ which, in most cases, cause a deficit in reasoning, memory and attention. It also occurs due to functional and morphological changes caused by the seizures,^9^ in association with injuries, aging and progressive brain damage.^10^ ^,^ ^11^ Therefore, deficits in cognition and memory are among the main concerns regarding epileptic patients, and overall, physicians tend to underestimate the importance of these conditions.^12^ ^,^ ^13^

It is known that some anticonvulsant medications, such as phenytoin, affect the quality of life of the epileptic patients by causing cognitive impairment.^14^ ^–^ ^17^ Furthermore, another study conducted with a series of neuropsychological tests to assess memory and psychomotor skills, showed that about 53% of newly diagnosed and untreated epileptic patients had at least abnormal scores in comparison to the mean of the control group.^15^ ^–^ ^17^

The purpose of this study was to demonstrate a non-inferiority test of MoCA in relation to the Mini Mental State Examination (MMSE) pursuant to improve screening strategies for the assessment of cognitive decline and memory disorders in patients with epilepsy. The evaluation was subdivided according to the etiology, lobe affected, type of seizures, and duration and use of medications, thus enabling to us to determine whether they promote effective assessments, contributing to the early recognition of cognitive deficits in epilepsy patients.

## METHODS

This study was observational and cross-sectional, whose participants were patients followed exclusively at an outpatient clinic of the Neurology Service Department, specialized in epilepsy, which is part of the tertiary healthcare level of the Brazilian Unified Health System.

The patients included were over 18 years old, of both genders, previously diagnosed with different types of epilepsy, and they also showed autonomy to answer the questionnaires, regardless of their socioeconomic and cultural differences. All patients gave consent to be included in the research.

The exclusion criteria consisted of: patients who, at the time of the appointment, refused to participate in this study; patients with hearing loss; patients with thyroid and/or liver disease; and patients previously diagnosed with mental retardation, depression, dementia or any other medical disorder that explains their cognitive deficits.

Two questionnaires, the MMSE and the Montreal Cognitive Assessment (MoCA), were answered by the patients, between June 2018 and March 2020. Also, during the interview, their personal information was obtained. The scales were applied by one investigator always on the same day (Monday) and order.

MMSE is a screening test already validated in the Portuguese language and used for previous studies in Brazil, which evaluates the cognitive functions in a simple and fast way. The cut-off point considered in our study was the same as proposed by Brucki et al.

MoCA is also a cognitive screening test, which is easy to perform. It evaluates cognitive domains such as memory, attention, concentration, executive functions, language, visuospatial abilities, capacity of abstraction, calculation and orientation. In this study, the addition of 1 point was maintained for those patients with less than 12 years of schooling.^7^ ^,^ ^11^

The results of quantitative variables were described by means, standard deviations, medians, and minimum and maximum values, while the categorical variables were described by frequencies and percentages. The non-parametric Mann-Whitney test was performed to compare two groups regarding the discrete quantitative variables. More than two groups were compared using the non-parametric Kruskal-Wallis test. A non-parametric approach was considered because of the type of variables (scores). Regarding the categorical variables, the comparisons were performed through either the Fisher exact test or chi-square test. The analysis of the correlation between two quantitative variables was then performed by estimating the Spearman correlation coefficient, and the normality of continuous quantitative variables was assessed by the Kolmogorov-Smirnov test. Receiver Operating Characteristic (ROC) curve analysis was performed, and the results obtained considered a % for specificity and a % for sensitivity [area under ROC curve p and 95% confidence interval (95%CI)]. Values of p<0.05 showed statistical significance.

The first part of this research was submitted in May 2018 to the Ethics Committee of the Plataforma Brasil and approved by the Associação Paranaense de Cultura from the Pontifícia Universidade Católica do Paraná (PUC-PR) and the second part was submitted in September 2019 and approved.

## RESULTS

### Demographic variables

The mean current age was 44.7 years and the mean diagnosis age was 30.8. Fifty percent of the individual were male. Regarding level of schooling, the majority went to elementary school (55.6%). All demographic variables are shown in Table 1.

**Table 1:** Demographic variables of the participants of the study (n=54).

Current age and age at the diagnosis						
	n	Mean	Median	Minimum	Maximum	Standard deviation
Current age (years)	54	44.7	44.5	19	78	14.6
Diagnosis age (years)	54	30.8	31	3	73	17.9

Sex and level of schooling			
	Classification	n	%
Biological sex	Male	27	50.0
	Female	27	50.0
Level of schooling	Preschool education^a^	2	3.7
	Elementary School^b^	30	55.6
	High School^c^	20	37.0
	Higher Education^d^	2	3.7
Level of schooling (Group)	Preschool/Elementary School	32	59.3
	High School/Higher Education	22	40.7

a Less than 9 years of study;

b 9 years of study;

c 12 years of study;

d a minimum period of 16 years.

### Clinical variables

The principal epilepsy etiology was structural lesions (51.9%). The types of seizures were approximately evenly distributed among generalized onset (29.6%), focal onset (37%) and focal to bilateral tonic-clonic (33.3%). Thirty individuals (55%) were characterized as refractory epilepsy. Each specific type of seizure and antiepileptic medication are shown in Table 2.

**Table 2:** Clinical variables of the participants of the study (n=54).

	Classification	n	%
Etiology	Structural	28	51.9
	Infectious	9	16.7
	Unknown	14	25.9
	Immune	1	1.9
	Metabolic	2	3.7
Type of seizures	Generalized onset	16	29.6
	Focal to bilateral tonic-clonic	18	33.3
	Focal onset	20	37.0
Non-motor autonomic	Yes	2	3.7
Non-motor sensory	Yes	3	5.6
Non-motor behavior arrest	Yes	9	16.7
Non-motor absence	Yes	2	3.7
Motor myoclonic	Yes	1	1.9
Motor tonic	Yes	2	3.7
Motor atonic	Yes	1	1.9
Motor clonic	Yes	0	0.0
Motor tonic-clonic	Yes	16	29.6
Antiepileptic medication	None	1	1.9
	Monotherapy	28	51.9
	Two or more	25	46.3
Antiepileptic medication (group)	None/monotherapy	29	53.7
	Two or more	25	46.3

### Questionnaires

In this study, the MMSE cut-off point was the same as the one proposed by Brucki et al. The mean of the MMSE score (max. 30) was 23.2±4.7 (10–30); immediate memory (0 to 3) was divided into 0 to 2 correct answers (16.7%, n=9) and 3 correct answers (83.3%, n=45), while the delayed recall memory (0 to 3) was divided into 0 or 1 correct answer (31.5%, n=17), 2 correct answers (35.2% n=19) and 3 correct answers (33.3% n=18).

The mean of the MoCA score (max. 30) was 17.2±5.4 (6–29); immediate memory (0 to 5) was divided into 0 to 3 correct answers (27.8%, n=15), 4 correct answers (35.2%, n=19) and 5 correct answers (37%, n=20), while delayed recall memory was divided into 0 correct answer (35.2%, n=19), 1 or 2 correct answers (37%, n=20) and 3 to 5 correct answers (27.8%, n=15).

### Mini Mental State Examination and Montreal Cognitive Assessment

**Figure 1:**
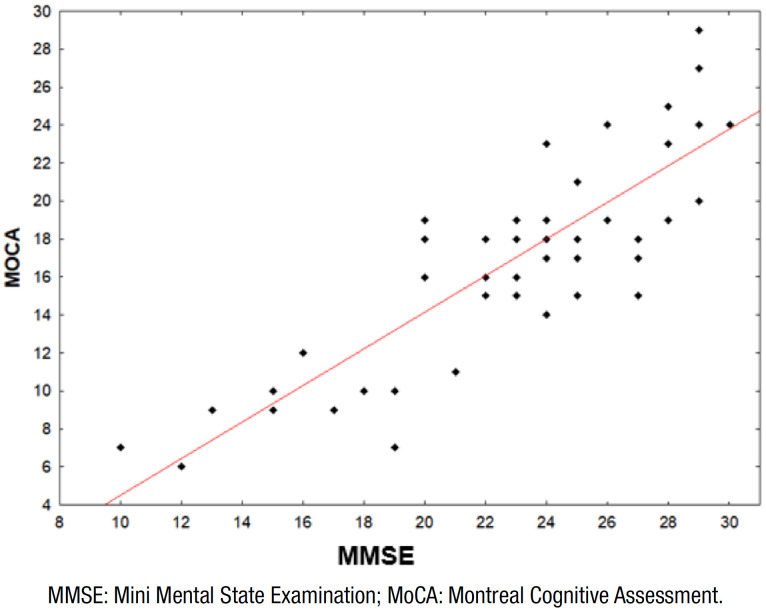
Spearman correlation coefficient=0.8.

We tested the null hypothesis, in which the correlation coefficient between the scores of MMSE and MoCA was equal to zero (no correlation), *versus* the alternative hypothesis, in which the correlation coefficient was different from zero (correlation). The estimated Spearman correlation coefficient was 0.80, with statistical significance (p<0.001). Thus, there was a significant correlation between the scores of both screening tests and there was no random error (Figure 1).

**Figure 2:**
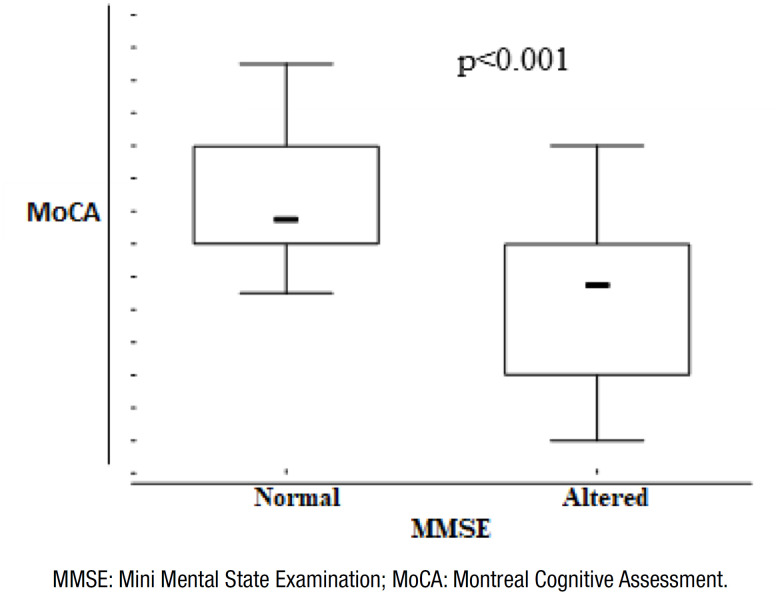
Mann-Whitney non-parametric test.

We tested the null hypothesis, in which the results of MoCA were the same for patients with normal MMSE scores as well as for patients with alterations in the MMSE scores, *versus* the alternative hypothesis of different results from Mann-Whitney non-parametric test. All 38 cases with low MMSE scores (according to the cut-off points and schooling) also showed low scores in the MoCA. In addition, among the 16 cases with normal MMSE, 14 (88%) had low scores in MoCA. During this analysis, a p<0.001 was observed, indicating the absence of random error and the presence of statistical significance (Figure 2).

**Figure 3:**
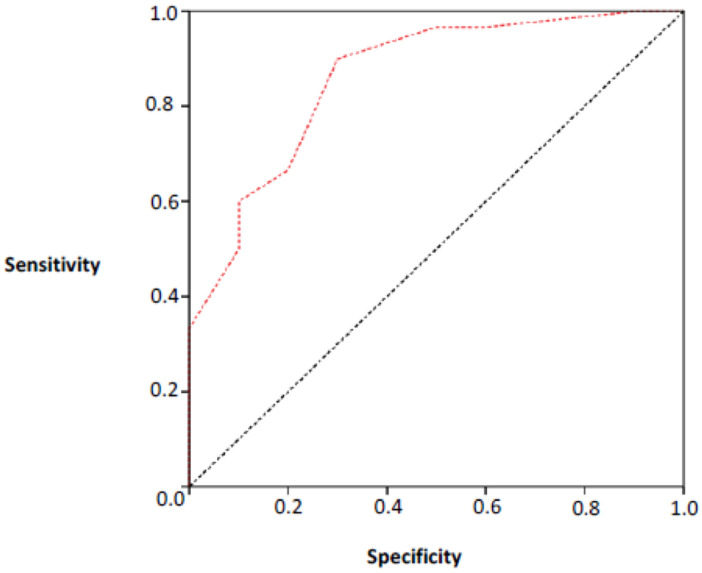
ROC Curve adjusted (area 0.82).

We also determined a cut-off point for the MoCA score that was associated with the MMSE result (normal or altered). For this analysis, a ROC curve for the MoCA scores was adjusted considering the results of the MMSE. The area under the curve was 0.82 with statistical significance (p=0.001). This indicates that the adjustment was good and that the MoCA score discriminated well between having normal or altered MMSE. The cut-off point for the MoCA score indicated by the adjustment is equal to 18. Therefore, scores above 18 are associated with normal MMSE and scores up to 18 were associated with altered MMSE. The sensitivity of this cut-off point was estimated to be 90% (Figure 3).

### Age

We tested the null hypothesis, in which the Spearman correlation coefficient between age and score was equal to zero (no correlation), for each of the MMSE and MoCA scores, as well as for both the current age (at the time that the questionnaire was answered) and the age at diagnosis; also, we tested the alternative hypothesis that the Spearman correlation coefficient was different from zero (correlation). The current age and the MMSE and MoCA scores were significantly correlated, with p=0.002 and p=0.005, respectively, indicating the absence of random error.

The Spearman correlation coefficient was used during this same statistical analysis. Current age and MMSE score gave the coefficient −0.41, while current age and MoCA score yielded the coefficient −0.38. The negative sign of this correlation coefficient indicates that low values for current age correspond to high scores in both MMSE and MoCA. The age at the epilepsy diagnosis was not significantly correlated (p>0.05).

### Schooling

We tested the null hypothesis, in which the results were equal for all the classifications regarding schooling, *versus* the alternative hypothesis of different scores (Table 3).

**Table 3:** Schooling × Mini Mental State Examination and Montreal Cognitive Assessment scores (n=54).

Score	Level of schooling	n	Mean	Median	Minimum	Maximum	Standard deviation	p-value*
MMSE	Preschool/elementary school	32	21.4	22.5	10	29	4.9	
	High school/higher education	22	25.9	26	20	30	2.7	**<0.001**
MoCA	Preschool/elementary school	32	14.6	15	6	25	4.7	
	High school/higher education	22	21.1	20	15	29	3.5	**<0.001**

MMSE: Mini Mental State Examination; MoCA: Montreal Cognitive Assessment;

* Mann-Whitney non-parametric test, p<0.05.

### Schooling × memory

We also tested the null hypothesis, in which there was no association between the factor and the variable, *versus* the alternative hypothesis, in which there was an association (Table 4).

**Table 4:** Schooling × immediate delayed recall memory (n=54).

	Classification	Level of schooling		p-value*
		Preschool/elementary school	High school/higher education	
MMSE immediate memory	0 to 2	6 (18.8)	3 (13.6)	
	3	26 (81.3)	19 (86.4)	0.723
MMSE delayed recall memory	0 to 1	13 (40.6)	4 (18.2)	
	2	11 (34.4)	8 (36.4)	
	3	8 (25)	10 (45.5)	0.154
MoCA immediate memory	0 to 3	11 (34.4)	4 (18.2)	
	4	12 (37.5)	7 (31.8)	
	5	9 (28.1)	11 (50)	0.219
MoCA delayed recall memory	0	17 (53.1)	2 (9.1)	
	1 to 2	11 (34.4)	9 (40.9)	
	3 to 5	4 (12.5)	11 (50)	**0.001**

MMSE: Mini Mental State Examination; MoCA: Montreal Cognitive Assessment;

* Fisher's exact test or chi-square test, p<0.05.

### Type of seizures × memory

We also tested the null hypothesis, in which there is no association between the factor and the variable, *versus* the alternative hypothesis, in which there is an association (Table 5).

**Table 5:** Type of epileptic seizure × immediate memory and delayed recall memory (n=54).

	Classification	Type of seizures			p-value*
		Generalized onset	Focal to bilateral tonic-clonic	Focal start	
MMSE immediate memory	0 to 2	1 (6.3)	3 (16.7)	5 (25)	
	3	15 (93.8)	15 (83.3)	15 (75)	0.325
MMSE delayed recall memory	0 to 1	2 (12.5)	6 (33.3)	9 (45)	
	2	6 (37.5)	9 (50)	4 (20)	
	3	8 (50)	3 (16.7)	7 (35)	0.081
MoCA immediate memory	0 to 3	1 (6.3)	7 (38.9)	7 (35)	
	4	4 (25)	7 (38.9)	8 (40)	
	5	11 (68.8)	4 (22.2)	5 (25)	0.030
MoCA delayed recall memory	0	3 (18.8)	7 (38.9)	9 (45)	
	1 to 2	7 (43.8)	9 (50)	4 (20)	
	3 to 5	6 (37.5)	2 (11.1)	7 (35)	0.128

MMSE: Mini Mental State Examination; MoCA: Montreal Cognitive Assessment;

* chi-square test, p<0.05.

## DISCUSSION

Memory is a set of brain systems that allow the processing of information for later use after a time interval consciously or not. Two types of memory: (1) hippocampal memory (declarative or episodic) is explicit or conscious evocation; (2) non-hippocampal memory (non-declarative or procedural) is implicit or unconscious evocation. Short-term memory is working memory (frontoparietal working memory) plus processing, storage, evocation for memory consolidation (hippocampal). Long-term memory is episodic or autobiographical (hippocampal), knowledge or semantic (mesial temporal lobe) and procedural (basal ganglia).

Several studies correlate the deficits of memory and cognition in epilepsy, mainly, to the temporal lobe epileptogenic focus. More specifically, memory issues are due to a lesion in the dominant medial temporal lobe, but there are reports regarding the extratemporal and frontal lobe epilepsy.^9^ All these types of epilepsy result in more serious comorbidities in comparison to the generalized seizures.^18^

The main objective of this study was to compare the screening tests for decline in cognition and memory through the MMSE and MoCA questionnaires. The MoCA was, in fact, created to be more sensitive to abnormal performance of cognitive domains, such as visuospatial, executive function, naming, attention, language, abstraction, delayed recall memory and orientation.^19^ Regarding memory, MoCA involves more words for the training of immediate and delayed recall memory, as well as more learning tasks; also, it has a longer time interval for recall in comparison to MMSE.

In our study, 38 patients scored below the cut-off points established by their level of schooling in the MMSE questionnaire, also obtaining low scores in MoCA. The results of the MMSE showed a difference of 14 patients, who had alteration only in MoCA scores, which categorized 52 patients with scores below expectations. Similar results were found in one study, regarding abnormal scores in epileptic patients (53.5%) from a series of neuropsychological tests.^20^ However, no patient who had a low score in MMSE was categorized as normal in MoCA in our study, which did occur in the aforementioned study.

There are several factors that directly or indirectly interfere with the cognitive performance of epilepsy patients, such as the frequency of seizures, the antiepileptic medication in use, age, and location of the epileptogenic focus.^21^ ^–^ ^23^ In this sense, a screening for cognitive impairment is very important. It remains unknown if the cause is the early onset of epilepsy, the accumulation of brain damage due to seizures or the interaction of an initial precipitating lesion with physiological or senile processes.^23^ ^,^ ^24^

In our study, 19 patients underwent monotherapy treatment, while 21 patients underwent treatment with a combination of several different drugs. Several reports have shown that antiepileptic drugs (AED) might be associated with adverse cognitive effects.^25^ Some authors claim that the control of seizures with monotherapy using first-line drugs is beneficial for cognitive functions, because of the reduction of the accumulated brain damage.^26^ ^,^ ^27^ Otherwise, limiting the number of AEDs should also be prioritized by clinicians.^23^

In this study, immediate memory evaluates the quality of the memory immediately after the presentation of the stimulus. The words used in the MMSE test were different from those used in the MoCA test. The delayed recall analyzed by the MOCA test showed a significant relationship with the patients’ level of schooling, which did not occur in the MMSE evaluation. This outcome can be explained by the two additional words for recall in MoCA totaling 5 new words to be memorized.

In our study, patients with focal-onset seizures had worse performance in immediate memory of MoCA (p<0.05). Similar results occurred in other studies that analyzed patients with complex focal seizures and used different tests that evaluated the same type of memory, such as that by Stella.^28^ The author studied mnemonic activity in epileptic patients with complex partial seizures through the Wechsler Memory Test. In the three subtests, the patients showed cognitive performance significantly lower than the controls (p<0.05).

We know that epilepsy patients can experience specific effects on memory, depending on the cause of the seizures and on their location. Memory deficits are associated with the extent and location of the damage in the brain structure, as well as with the degree of physiological dysfunction, the frequency and severity of seizures, the neurotoxicity of antiepileptic drugs and the degree of cognitive development at the onset of diagnosis.^29^

Cognitive deterioration varies with the laterality affected by the epileptogenic focus, since the involvement of the left lobe causes deficiencies in verbal memory, while that of the right lobe causes deficiencies in non-verbal memory.^24^ The duration of the seizures was considered another impact factor in the cognition of these individuals, exemplified by prolonged epileptic seizures of 30 minutes or more.^30^ ^–^ ^32^

There are similar items in both questionnaires; however, these same questions were not repeated a second time. The entire methodology was well designed and executed. The questions that appeared in the MMSE and had an equivalent answer in the MoCA were only asked once, during the first application (MMSE). In conclusion, all items in the topic “Orientation” in MoCA are contained in the topic “Orientation” in MMSE, and thus, the patient was not asked these items again. Same as subtraction contained in “Attention”. Hence, we believe that there was no response bias or information bias in our study. However, this study had some limitations, such as its design, which is cross-sectional and allows the evaluation of the cognitive domains at only one moment. Another point is the fact that tertiary center patients may have a worse epilepsy condition in comparison with the general population.

The present study was able to identify alterations in patients with an MMSE considered normal, through the performance of the MoCA screening test. None of the patients showed a low MMSE score with a normal MoCA score. Therefore, we found MoCA to have a superior accuracy as a screening test, so we encourage the use of MoCA because it has greater specificity (less false-negatives). Furthermore, we emphasize the importance of memory screening tests in epileptic patients to differentiate cognitive complaints from normal aging. The utilization of such tools regularly in large studies or primary care, could identify the need for a more complete cognitive analysis.

Regarding the division into subgroups, we observed a significant relationship between immediate and delayed recall memory and the type of seizures and schooling, respectively. Thus, it becomes relevant to add this screening test in the evaluation of cognition and memory in epilepsy patients.

But what can we do with this information? Neuroplasticity is very important in this context. “Neurons that fire together wire together” is understood as the capacity of the brain (neurons and neural networks) to reorganize and change itself to compensate injury or dysfunction.^33^

Hippocampal neurogenesis (in the dentate gyrus) is changed by brain injury (such as in epilepsy), but hippocampal neurogenesis is also changed (and this time for good) by aerobic physical exercise, slow-wave sleep, chronic treatment with antidepressants plus cognitive training.^34^

If there is one major takeaway from this research, it is that the use of original articles, guidelines and consensus to aid decision-making improves clinical management, contributes to a change in the organizational culture of the medical class and strengthens evidence-based medicine. Whether or not the results hold up to scrutiny, we think that however small this study is and with its limitations, it may contribute to the implementation of effective strategies for screening for cognitive decline and memory in patients with epilepsy.
